# Supplementary material for: Buccal Resveratrol Delivery System as a Potential New Concept for the Periodontitis Treatment
Source: Pharmaceutics. 2021 Mar 20;13(3):417. doi: 10.3390/pharmaceutics13030417 (PMC8003728; doi:10.3390/pharmaceutics13030417)
Supplement: Supplementary file 1 [file pharmaceutics-13-00417-s001.pdf]

pharmaceutics

MDPI

Supplementary Material: Buccal Resveratrol Delivery System as a Potential New Concept for the Periodontitis Treatment

Magdalena Paczkowska-Walendowska, Jakub Dvořák, Natalia Rosiak, Ewa Tykarska, Emilia Szymańska, Katarzyna Winnicka, Marek A. Ruchała and Judyta Cielecka-Piontek

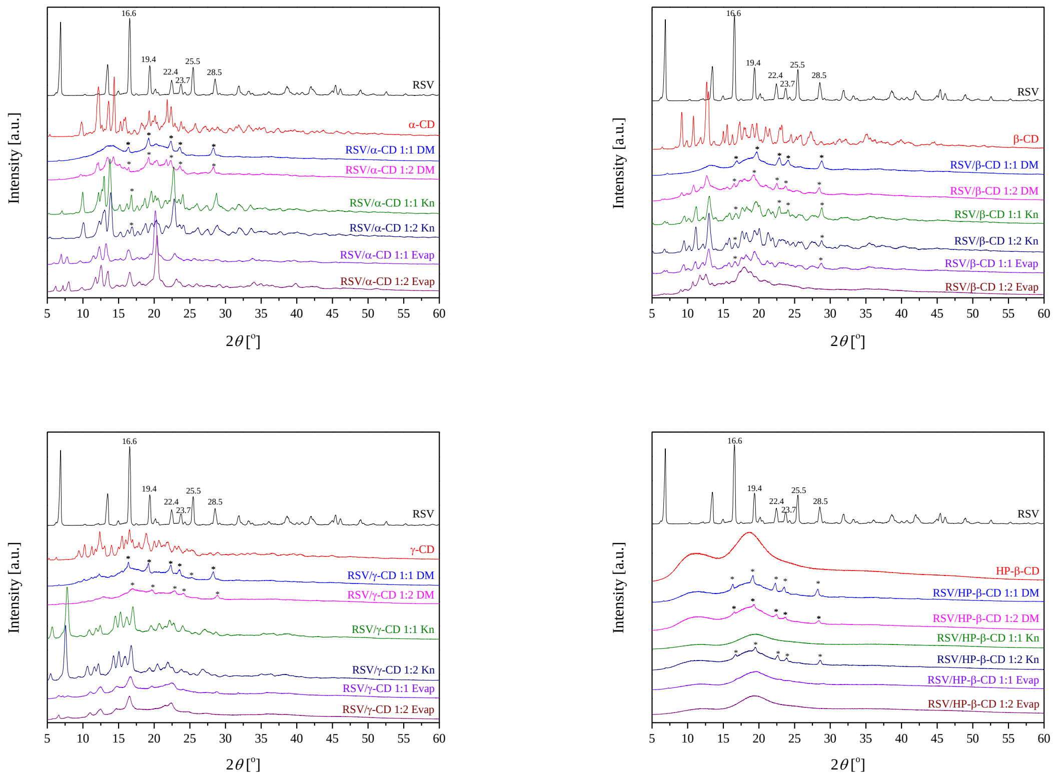

Figure S1. X-ray diffractograms of the solid samples. 2θ positions of the principal diffraction peaks are shown for RSV, while the traces of crystalline RSV peaks in the systems are indicated by "*".

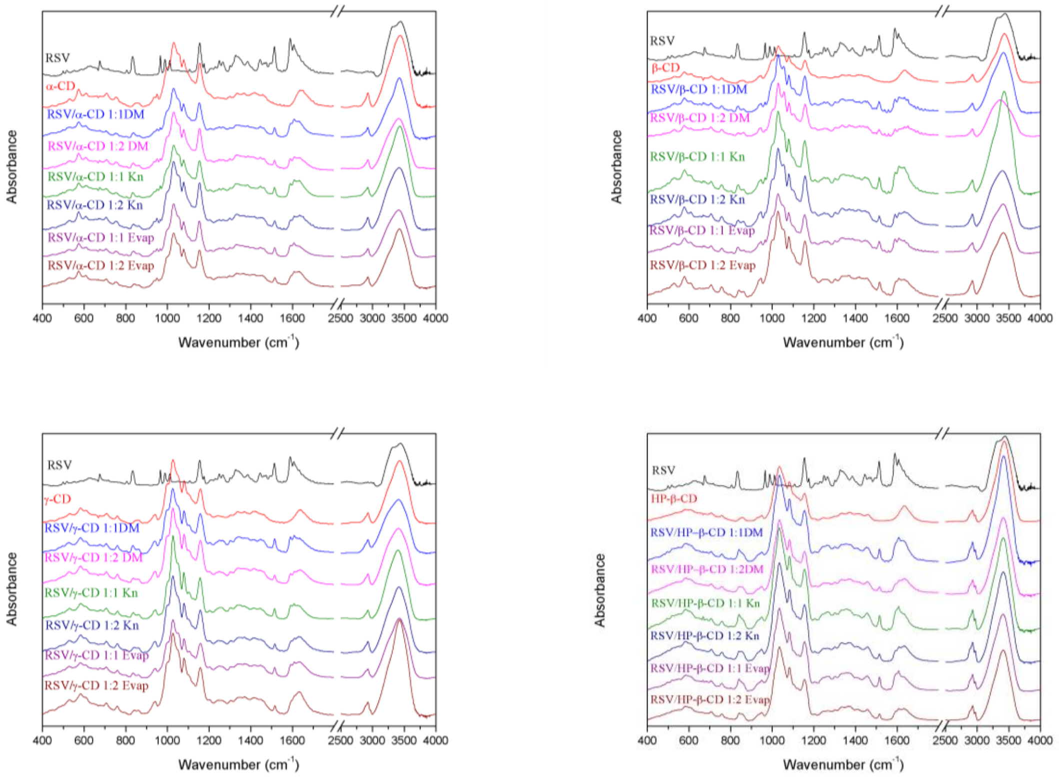

Figure S2. FT-IR spectra of powder RSV/CD systems.

Table S1. Validation parameters of the HPLC-DAD method.

Parameter	Results
Linearity: y = *a*x + *b*	
$a \pm S_a$	54 072.50 ± 2.00
$b \pm S_b$	insignificant (α=0.05)
Correlation coefficient (*r*)	0.9999
Range of linearity [mg mL^{-1}]	0.1–40.0
Intra-day precision, RSD (<5% required) = repeatability	
0.4 [mg mL^{-1}]	0.57
4.0 [mg mL^{-1}]	0.11
40.0 [mg mL^{-1}]	0.14
Inter-day precision = reproducibility	
0.4 [mg mL^{-1}]	0.52
4.0 [mg mL^{-1}]	1.09
40.0 [mg mL^{-1}]	0.79
Limit of detection (LOD) [µg mL^{-1}]	1.81
Limit of quantification (LOQ) [µg mL^{-1}]	5.48

S_a standard deviation of slope; S_b standard deviation of intercept, *t*, calculated values of the Student's t test, $t_{\alpha,f}$ = 2.228 critical values of the Student's test for degrees of freedom f = 10 and significance level α = 0.05.

Table S2. The antioxidant effect of RSV/CD powder systems.

	**DPPH** **IC_{50} [µg mL^{-1}]**	**CUPRAC** **[$IC_{0.5}$ µg mL^{-1}]**
RSV	22.1	33.2
RSV/α-CD 1:1 DM	21.1	32.5
RSV/α-CD 1:2 DM	21.1	32.5
RSV/α-CD 1:1 Kn	25.0	26.3
RSV/α-CD 1:2 Kn	25.6	26.5
RSV/α-CD 1:1 Evap	24.5	26.4
RSV/α-CD 1:2 Evap	24.5	25.5
RSV/β-CD 1:1 DM	20.3	25.0
RSV/β-CD 1:2 DM	20.9	25.8
RSV/β-CD 1:1 Kn	21.0	26.7
RSV/β-CD 1:2 Kn	21.3	26.3
RSV/β-CD 1:1 Evap	27.1	30.1
RSV/β-CD 1:2 Evap	23.6	25.8
RSV/HP-β-CD 1:1 DM	26.7	33.1
RSV/HP-β-CD 1:2 DM	24.3	33.1
RSV/HP-β-CD 1:1 Kn	26.5	31.1
RSV/HP-β-CD 1:2 Kn	25.1	34.6
RSV/HP-β-CD 1:1 Evap	27,5	33.2
RSV/HP-β-CD 1:2 Evap	21.9	30.1
RSV/γ-CD 1:1 DM	22.2	24.8
RSV/γ-CD 1:2 DM	21.9	24.3
RSV/γ-CD 1:1 Kn	20.3	27.2
RSV/γ-CD 1:2 Kn	21.3	25.5
RSV/γ-CD 1:1 Evap	18.2	23.7
RSV/γ-CD 1:2 Evap	20.3	25.8
F1	20.6	31.6
F2	23.1	28.7
F3	23.1	26.5
F4	22.4	32.3
F5	24.0	32.5

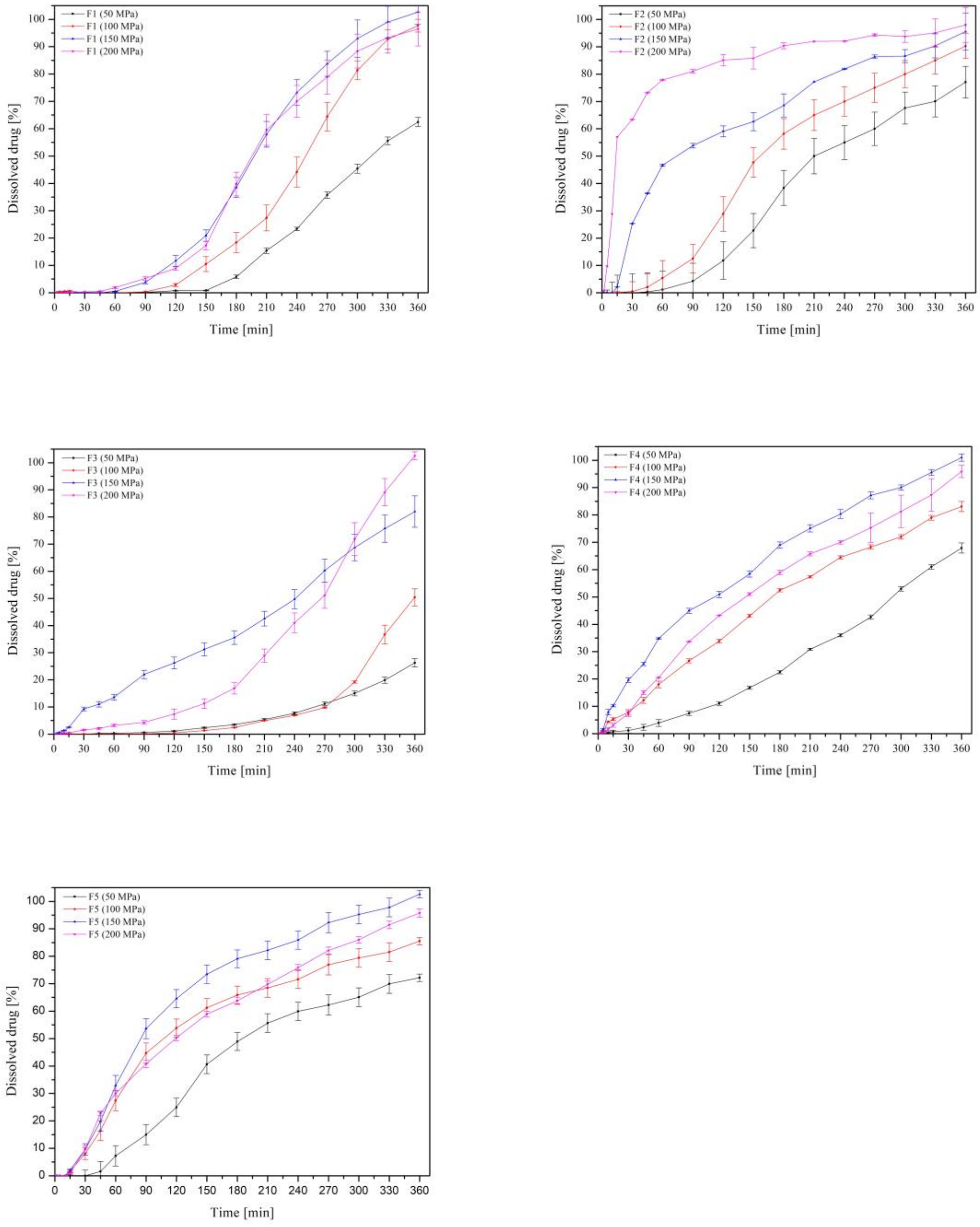

Figure S3. Dissolution profiles of the RSV/CD tablets (F1-F5).

Table S3. Mathematical characteristics of the RSV/CD tablets (F1-F5)

Formulation	Mathematical model								
	Zero-order kinetic		First-order kinetic		Higuchi kinetic		Korsmeyer-Peppas kinetic		
	K	R^2	K	R^2	K	R^2	K	R^2	n
F1 (7.5 kN)	18.99	0.**9474**	1.0396	0.7715	6.3796	0.8058	1.8203	0.6785	0.3542
F1 (10 kN)	17.982	0.**944**	1.0711	0.8658	6.0566	0.8065	2.0376	0.7895	0.3716
F2 (7.5 kN)	16.23	0.8835	0.7931	0.4958	6.3349	0.**9534**	0.7005	0.6532	0.339
F2 (10 kN)	12.732	0.5988	0.4198	0.327	5.9132	0.**8268**	1.1829	0.6022	0.2357
F3 (7.5 kN)	13.391	0.**9939**	0.713	0.764	4.7674	0.9253	0.5358	0.9077	0.2953
F3 (10 kN)	15.048	0.8756	0.8841	0.**9571**	4.9664	0.7237	1.3939	0.9158	0.3181
F4 (7.5 kN)	16.998	0.9595	0.6834	0.5208	6.4357	0.**9792**	0.2138	0.7395	0.3105
F4 (10 kN)	16.428	0.**9808**	0.7584	0.6948	6.0052	0.9532	0.5816	0.8573	0.3189
F5 (7.5 kN)	18.707	0.9134	0.7535	0.6697	7.0601	0.**9379**	0.4167	0.8461	0.3229
F5 (10 kN)	16.927	0.9552	0.8368	0.5914	6.3114	0.**9593**	0.9099	0.7259	0.3446

The highest R^2 values in bold.
